# [Corrigendum] Mesenchymal stem cells expressing interleukin‑18 inhibit breast cancer in a mouse model

**DOI:** 10.3892/ol.2023.13986

**Published:** 2023-07-31

**Authors:** Xiaoyi Liu, Jianxia Hu, Yueyun Li, Weihong Cao, Yu Wang, Zhongliang Ma, Funian Li

Oncol Lett 15: 6265–6274, 2018; DOI: 10.3892/ol.2018.8166

Subsequently to the publication of the above paper, an interested reader drew to the authors’ attention that, for the histology experiments shown in Fig. 5A on p. 6271, the ‘hUMSCs’ and ‘hUMSCs/vector’ panels appeared to show overlapping sections of data, such that they were likely to have been derived from the same original source where the results of differently performed experiments were intended to have been shown.

The authors have re-examined their data and realized that Fig. 5A was inadvertently assembled incorrectly; however, the authors still had access to their original data, and the revised version of Fig. 5, now including the correct data panel for the ‘hUMSCs/vector’ experiment, is shown opposite. The authors regret the error that was made during the preparation of this figure, although they were able to confirm that this did not seriously affect the conclusions reported in the paper. The authors are grateful to the editor of *Oncology Letters* for allowing them the opportunity to publish a Corrigendum, and all the authors agree to this Corrigendum. Furthermore, they apologize to the readership for any inconvenience caused.

## Figures and Tables

**Figure 3.**
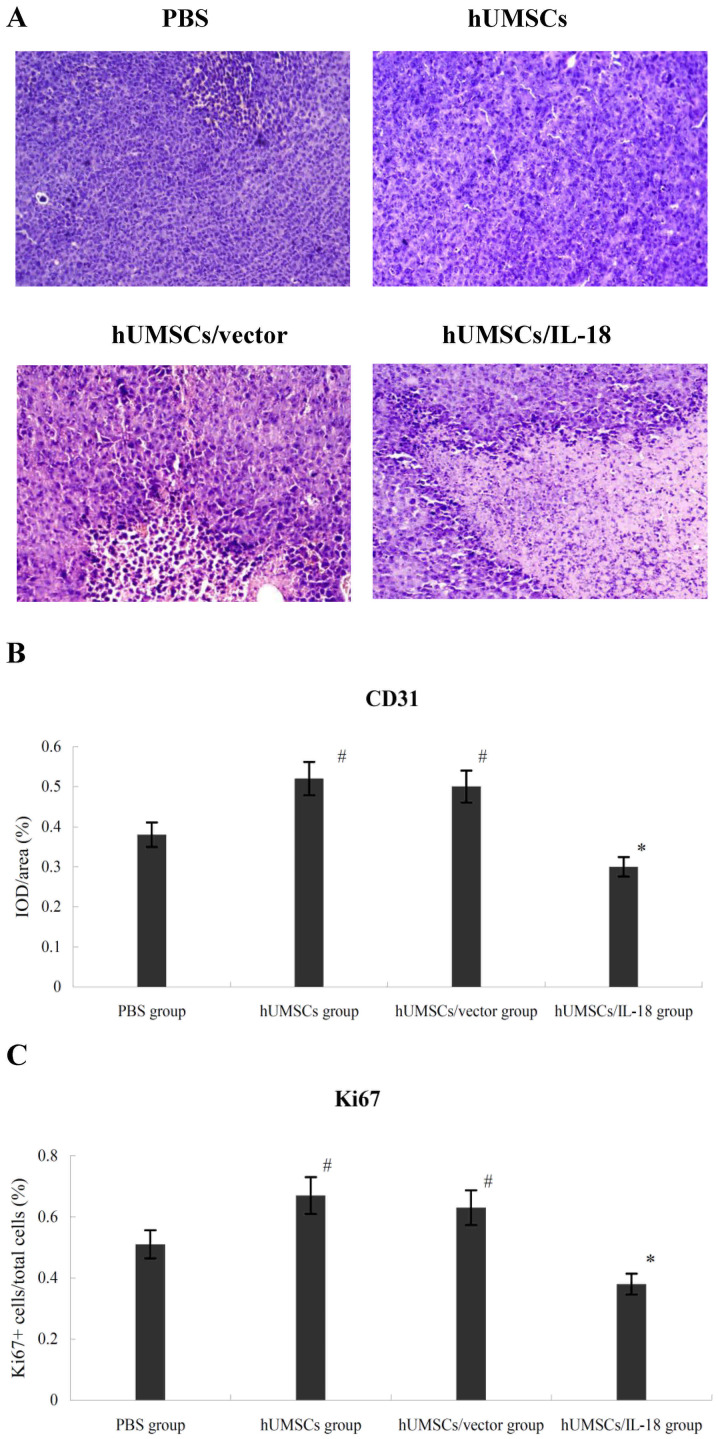
GA inhibits the expression of Ki-67 in the NCI-H1993 ×enograft model. The NCI-H1993 ×enograft model was treated with various doses of GA, vehicle or positive control for 2 h on day 21 of the efficacy study. (A) Representative images of Ki-67 staining. (B) Quantification of Ki-67 positive area (%). Values are expressed as the mean ± standard deviation, n=10. *P<0.05 vs. vehicle group; **P<0.01 vs. vehicle group. GA, gambogic acid; i.p., intraperitoneal; i.v., intravenous.

